# Curcumin inhibits EMMPRIN and MMP-9 expression through AMPK-MAPK and PKC signaling in PMA induced macrophages

**DOI:** 10.1186/s12967-014-0266-2

**Published:** 2014-09-21

**Authors:** Jiatian Cao, Zhihua Han, Lei Tian, Kan Chen, Yuqi Fan, Bozhi Ye, Weijian Huang, Changqian Wang, Zhouqing Huang

**Affiliations:** Division of Cardiology, Shanghai Ninth Peoples Hospital Affiliated Shanghai Jiaotong University School of Medicine, Shanghai, P. R. China; Division of Cardiology, the First Affiliated Hospital of Wenzhou Medical University, Wenzhou, Zhejiang P. R. China

**Keywords:** Curcumin, EMMPRIN, MMP-9, MMP-13, AMPK, MAPK, Atheroslerosis

## Abstract

In coronary arteries, plaque disruption, the major acute clinical manifestations of atherosclerosis, leads to a subsequent cardiac event, such as acute myocardial infarction (AMI) and unstable angina pectoris (UA). Numerous reports have shown that high expression of MMP-9 (matrix metalloproteinase-9), MMP-13 (matrix metalloproteinase-13) and EMMPRIN (extracellular matrix metalloproteinase induce) in monocyte/macrophage results in the plaque progression and destabilization. Curcumin exerts well-known anti-inflammatory and antioxidant effects and probably has a protective role in the atherosclerosis. The purpose of our study was to investigate the molecular mechanisms by which curcumin affects MMP-9, MMP13 and EMMPRIN in PMA (phorbol 12-myristate 13-acetate) induced macrophages. Human monocytic cells (THP-1 cells) were pretreated with curcumin or compound C for 1 h, and then induced by PMA for 48 h. Total RNA and proteins were collected for real-time PCR and Western blot analysis, respectively. In the present study, the exposure to curcumin resulted in attenuated JNK, p38, and ERK activation and decreased expression of MMP-9, MMP-13 and EMMPRIN in PMA induced macrophages. Moreover, we demonstrated that AMPK (AMP-activated protein kinase) and PKC (Protein Kinase C) was activated by PMA during monocyte/macrophage differentiation. Furthermore, curcumin reversed PMA stimulated PKC activation and suppressed the chronic activation of AMPK, which in turn reduced the expression of MMP-9, MMP-13 and EMMPRIN. Therefore, it is suggested that curcumin by inhibiting AMPK-MAPK (mitogen activated protein kinase) and PKC pathway may led to down-regulated EMMPRIN, MMP-9 and MMP-13 expression in PMA-induced THP-1 cells.

## Background

EMMPRIN, also termed CD147 or M6 antigen, is a 58-kDa cell surface glycoprotein described first in tumor cells. It participates in numerous physiological processes, play a central role in tumor metastasis, cell adhesion, angiogenesis, chemoresistance and atherosclerosis [1,2]. EMMPRIN has been reported to stimulates secretion of MMP-9 (matrix metalloproteinase-9) in monocytes [1,3], have strong positive correlation with MMP13 [4,5] or several MMPs in other cells [6,7], and activates MMP-9 in atherosclerotic plaque [8]. MMP-9 belongs to a family of zinc- and calcium-dependent endopeptidases. It is a 92 kDa protein that regulates numerous cell activities, involving in various physiological functions, such as cell-cell contact, tissue remodeling cell migration and cellular differentiation [9]. Recent data showed that increased EMMPRIN expression affects plaque stability [1,8], and accelerates the transition from a stable plaque to an unstable plaque in atherogenic cells, such as monocytes/macrophages and coronary smooth muscle cells [10,11]. Despite recent advance in drug treatment and surgical therapies, atherosclerosis remains to be a major cause of death throughout the world. In coronary arteries, plaque disruption is the majority of acute clinical manifestations of atherosclerosis, leading to a subsequent cardiac event, such as AMI and UA. Monocyte-derived macrophages are known to play a critical role in the initiation and progression of atherosclerosis. Over-expression of MMP-9 and EMMPRIN in monocytes/macrophages results in plaque progression and destabilization [6,12]. Plaque rupture is thought to result from the degradation of extracellular matrix components by macrophage-derived matrix metalloproteinases (MMPs) [13]. Numerous reports have shown that MMP-9 is one of the most important MMPs contributing to plaque rupture, and its expression level is induced in serious coronary atherosclerosis and AMI and UA [14]. In addition, MMP-9 induces acute plaque disruption in Apoe−/−mice [15,16]. Previous reports demonstrated that MMP-13 is involved in atherogenesis and decreasing plaque stability [17]. MMP-13 might be overexpressed in both human and experimental atherosclerosis as well [18,19]. All these data indicate that EMMPRIN-mediated MMPs induction is involved in the process of atherosclerotic lesion. Base on these pieces of evidence, we hypothesized that agents suppressing EMMPRIN and MMP-9 expression would be potential therapeutic agents that ameliorate the development of atherosclerosis. All these data indicate that EMMPRIN-mediated MMP induction is involved in the process of atherosclerotic lesion. Based on these pieces of evidence, we hypothesized that agents suppressing EMMPRIN and MMP-9 expression would be potential therapeutic agents that ameliorate the development of atherosclerosis.

During past few years, accumulating evidence has suggested that curcumin has significant inhibitory effect on MMPs in cancer, arthritis and ulcer [20]. Curcumin (diferuloylmethane), a polyphenol derived from turmeric and curcuma longa, is a pharmacologically safe and effective agent that plays an important role in anti-cancer and anti-inflammatory processes. In atherosclerosis, curcumin suppresses oxLDL (oxidized low-density lipoprotein) induced CD36 expression via inhibiting p38 MAPK phosphorylation [21], and prevents the decrease of thrombospondin-4 expression in oxLDL treated murine macrophages [21]. Curcumin inhibits the adhesion of monocytes to endothelial cells [22], and reduces the migration of HASMCs (human aortic smooth muscle cells) by suppressing MMP-9 expression through down-regulation of NF-κB -dependent pathways [23]. Furthermore, in vivo data showed that curcumin inhibits atherosclerosis in ApoE(−/−) mice [22], and blocks the development of atherosclerosis in ApoE/LDLR−/−mice [24]. Although some studies have suggested the anti-atherosclerosis activity of curcumin, the mechanism by which curcumin regulates MMP-9, MMP-13 and EMMPRIN is currently unknown. The purpose of this study was to uncover the mechanism by which curcumin regulates EMMPRIN, MMP-9 and MMP-13expression during monocyte differentiation.

## Materials and methods

### Cell culture

Human monocytic cell line THP-1 was obtained from American Type Culture Collection (ATCC, Rockville, MD, USA) and maintained at a density of 10^6^/ml in RPMI 1640 medium containing 10% FBS, 10 mM HEPES (Sigma) and 1% pen/strep solution at 37°C, 5% CO_2_ incubator. Cells were cultured in six-well plates for 48 h in the presence of 100 nM PMA, which allowed them to differentiate into adherent macrophages [25]. Cells were pretreated with curcumin (0 to 50 μM, Sigma, USA) or 10 μM Compound C (AMPK inhibitor), PD98059(MAPKK inhibitor), SB203580(p38 MAPK inhibitor), and SP600125(JNK inhibitor) MAP kinase inhibitors (Sigma, USA) for 1 hour, and then stimulated with PMA for another 48 hours.

### Cytotoxicity assay

PMA-induced macrophages were seeded in 96-well plates at 6 × 10^3^ cells/well. Twenty four hours later, cells were incubated with curcumin (0 to 100 μM) for 48 h. Cells without any treatment were used as a control. CCK8 assay (WST-8, Dojindo, Kumamoto, Japan) was used to assess the cytotoxicity of curcumin on PMA-induced macrophages, based on the manufacturer's recommendation.

### Protein isolation and Western blot analysis

Protein isolation and Western blot analysis of cell lysates were performed as previously described [26]. Briefly, membranes were first probed with primary antibodies for MMP-13(Proteintch,USA), EMMPRIN, PKCα, PKCβ1 (Life Technologies, USA), MMP-9, phospho-ERK, ERK, phospho-p38, p38, phospho-JNK, JNK, AMPK, p-AMPKα (Cell Signaling Technology, Boston, MA) (1:1000 dilution in TBST), or β-actin (1:5000 dilution in TBST), then incubated with anti-Rabbit or anti-mouse secondary antibodies (Cell Signaling Technology, Boston, MA), followed by incubation with antibody labeled with far-red fluorescent Alexa Fluor 680 dye. All signals were detected by the Odyssey imaging system (Li-cor, USA) and data were normalized based on the β-actin level.

### RNA isolation, cDNA synthesis and real-time PCR

Total RNA was extracted from PMA-induced macrophages using Trizol reagent (Invitrogen) according to the manufacturer's instructions. cDNA was synthesized using the Reverse Transcription Kit (Takara) before Real-time polymerase chain reactions were performed by SYBR Premix Ex Taq Kit (TaKaRa Code DRR041) according to the instructions . The PCR reactions were performed in duplicate and detected by the ABI-7500 Sequence Detection System (USA). The primer sequences are listed in Table 1. All results were normalized against the GAPDH level.

**Table 1:** **Primers used in realtime-PCR to measure the mRNA expression of MMP-9, MMP-13 and EMMPRIN**

**Primer for realtime-PCR**		
**Genes**	**Forward (5’-3’)**	**Reverse (5’-3’)**
MMP-9	TGACGCCGCTCACCTTCACT	CGCGCCATCTGCGTTTCCAA
MMP-13	CATTTGATGGGCCCTCTGGCCTGC	GTTTAGGGTTGGGGTCTTCATCTC
EMMPRIN	TTGGAGGTTGTAGGACCGGCGA	TGGGACCCTGCCCTTCAAACCA
GAPDH	CCGCATCTTCTTTTGCGTCGCC	TCTCAGCCTTGACGGTGCCA

### Gelatin zymography

Cells in the logarithmic phase were seeded in 6-well plate at the density of 3 × 10^5^ cells per well. After incubated in serum-free medium with or without curcumin (6.25, 25 and 50 μM) for 1 hour, cells were incubated with 100 μM PMA for another 48 h. culture supernatants were collected, 10 μl aliquots of the culture supernatant were loaded onto a 10% polyacrylamide gel containing 1 mg/ml gelatin. After electrophoresis, gels were washed twice with 2.5% Triton X-100 (37°C, 15 min) and then gels were incubated at 37°C for 11 h in developing buffer containing 10 mM Tris Base, 40 mM Tris–HCl, 200 mM NaCl, 10 mM CaCl2, 0.02% Brij 35. Gels were subsequently stained with 0.5% (w/v) Coomassie Blue R-250 for 2 h followed by destaining with a solution containing 50% methanol, 10% glacial acetic acid, 40% water. MMP-9-digested regions were visualized as light bands against a dark background. An image of each gel was detected by an Odyssey imaging system (Li-cor, USA).

### Statistical analysis

Data were presented as mean ± S.D and analyzed by one-way ANOVA. *P* < 0.05 was considered statistically significant. All experiments were performed at least three times.

## Results

### The cytotoxicity effect of curcumin on cells

**Figure 1:**
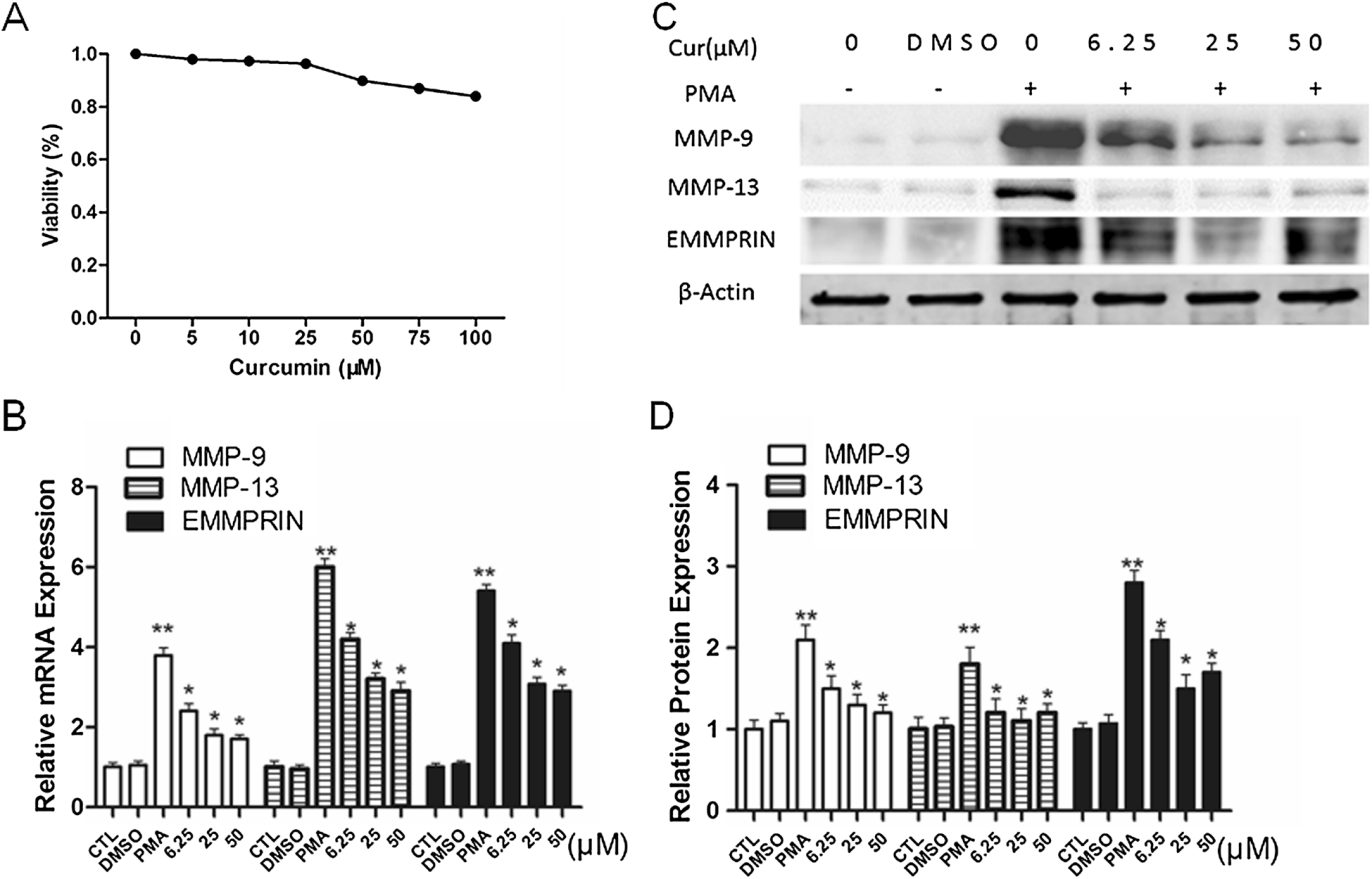
**Curcumin suppresses MMP-9, MMP13 and EMMPRIN expression in PMA induced macrophages. A**. Effect of curcumin on cell viability. PMA-induced macrophages were treated with indicated concentrations of curcumin (5–100 μM) for 48 h, and cell viability was assessed using the CCK-8 assay. Cells incubated in a medium without curcumin were defined as a control and were considered 100% viable. **B**-**D**. Curcumin suppresses EMMPRIN, MMP-9 and MMP13 expression in PMA-induced macrophages. The mRNA level and protein level were measured by Real-time PCR and Western blot, respectively. *P < 0.05 vs PMA group, **P < 0.05 vs CTL group. CTL indicates that cells incubated in a medium without curcumin. 6.25 μM, 25 μM, 50 μM indicates that THP-1 cells were pretreated with indicated concentrations of curcumin before stimulating with 100 nM PMA for another 48 h. Cur: curcumin.

To evaluate the cytotoxicity of curcumin on PMA-induced macrophages, cells were treated with 5, 10, 25, 50, 75 and 100 μM curcumin for 48 h, and then cell viability was detected by CCK-8 assay. As shown in Figure 1A, low-dose curcumin (≤50 μM) did not significantly (<10%) affect the cell viability. Therefore, cells were treated with dose less than 50 μM for no more than 48 hours in subsequent experiments.

### Curcumin reduces MMP-9, MMP13 expression and MMP-9 activity

**Figure 2:**
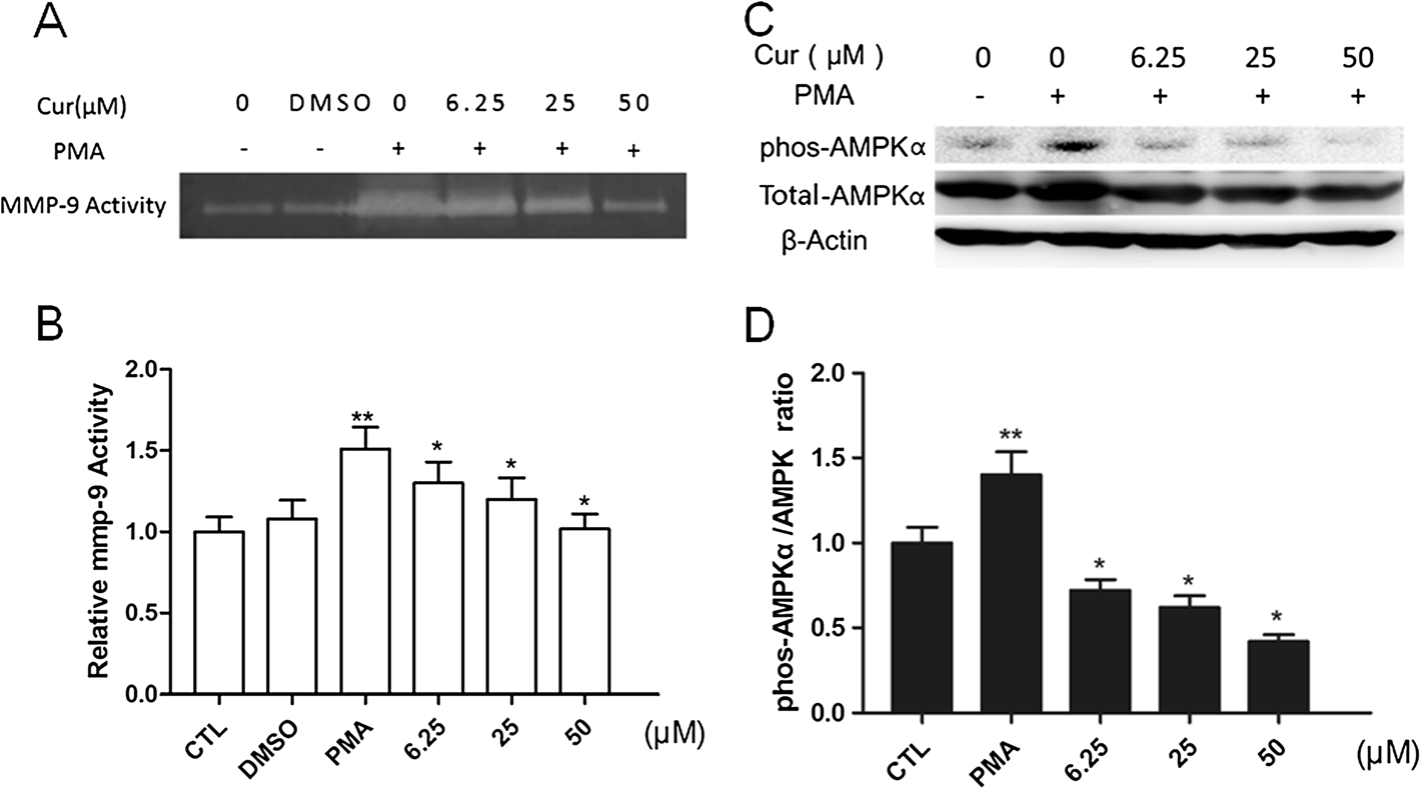
**Curcumin suppresses the activation of MMP-9 and chronic AMPK activation. A**-**B**. Effect of curcumin on the activity of MMP-9 from cultured supernatant. THP-1 cells were pretreated with curcumin before stimulating with PMA (100 nM) for another 48 h. MMP-9 activities were detected by gelatin zymography assay **(A)**, and the corresponding densitometric measurement was shown in **B**. Cur: curcumin. **C**-**D**. Effect of curcumin on total AMPK and phospho-AMPKα (Thr172). Differentiated THP-1 cells were treated with indicated agents and assayed by Western blot using indicated antibodies **(C)**, and the corresponding densitometric measurement was shown in **D**. *P < 0.05 vs PMA group, **P < 0.05 vs CTL group.

Elevated MMP-9 expression level was previously reported during the monocyte differentiation to macrophages, while MMP-13 expression level was unknown. To determine whether curcumin has any effect on MMP-9 and MMP-13 during the cell differentiation, THP-1 cells were pretreated with the indicated concentration of curcumin for 1 h, followed by incubating with 100 nM PMA for 48 h. Our results showed that curcumin significantly inhibited the upregulation of MMP-9 and MMP-13 induced by PMA, at both protein and mRNA levels, in a dose-dependent manner (Figure 1B-D). Because MMP-9 is reported to remarkably enhance elastin degradation in vitro and induce plaque rupture in vivo [15,27], we examined the effect of curcumin on MMP-9 enzymatic activity by SDS-polyacrylamide gelatin zymography assay. As previously reported, after overnight in-gel digestion, the gelatin-containing gel stained with coomassie blue showed an unstained transparent band at approximate 92 KDa, which was corresponding to the theoretical size of gelatin digested by MMP-9 [28]. In THP-1-derived macrophages, curcumin inhibited MMP-9 activity in a dose-dependent manner, as evidenced by gelatin zymography assay (Figure 2A,B). All the above data suggested that curcumin reduced MMP-13, MMP-9 expression and MMP-9 activity in a dose-dependent manner.

### Curcumin reduces EMMPRIN expression in a dose-dependent manner

EMMPRIN is the major and most characterized cell surface regulator of MMP-9 and MMP-13 production [11,12,18,19]. Since curcumin apparently down-regulates MMP-9 and MMP-13 expression in PMA-induced macrophages, we next tested whether the inhibitory effect of curcumin on MMP-9 and MMP-13 expression was due to the inhibition of EMMPRIN expression in PMA-induced macrophages. Indeed, our results showed that EMMPRIN expression was suppressed by curcumin in a dose-dependent manner at both protein and mRNA level (Figure 1B-D), suggesting that the down-regulation of EMMPRIN by curcumin is, at least in part, responsible for the reduction of MMP-9 expression in PMA-induced macrophages.

### Curcumin inhibits chronic AMPK activation induced by PMA

We further tested whether AMPK activation was involved in inhibiting MMP-9 and EMMPRIN expression by curcumin. Cells were pretreated with different doses of curcumin for 1 hour and induced with PMA for another 48 hours, then the phosphorylation of AMPKα and total AMPKα was examined by Western blot. As shown in Figure 2C-D, the total AMPK increased slightly in the PMA group and curcumin can attenuates upregulation of total AMPK protein.PMA induced the sustained activation of AMPKα in THP-1 cells. Importantly, curcumin remarkably abolished AMPKα activation in a dose-dependent manner.

### Curcumin suppresses MAPK and PKC pathways in PMA -induced THP-1 cells

**Figure 3:**
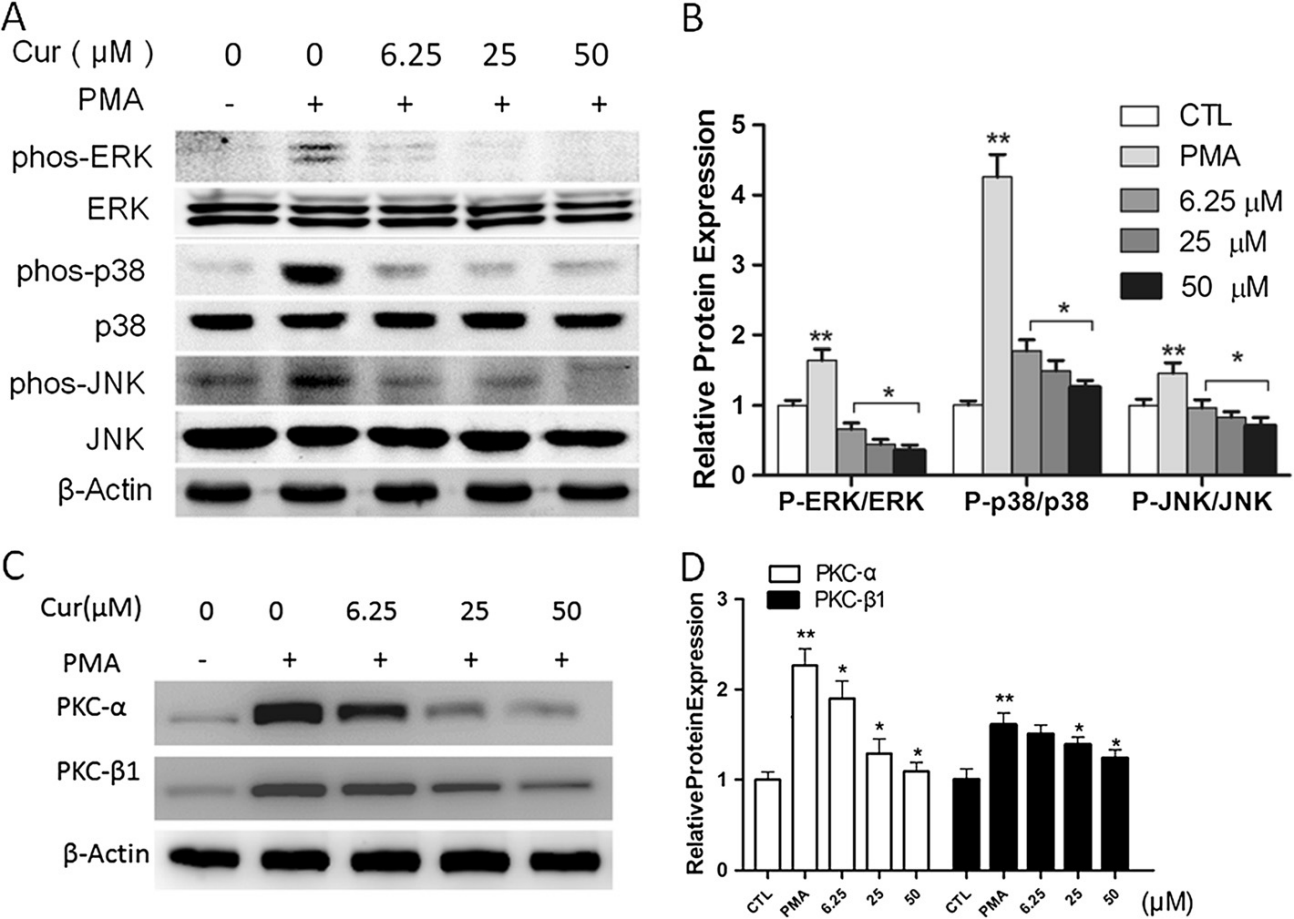
**Curcumin inhibits JNK, ERK, and p38 phosphorylation and PKC pathway. A**. Differentiated THP-1 cells were treated with indicated agents, and assayed by Western blot using indicated antibodies. Cells were pretreated with vehicle or curcumin at the indicated concentration (5–50 μM) for 1 h, followed by PMA for 48 h. **B**. Protein quantification was carried out by densitometric analysis. Normalized proteins of JNK, p-JNK, ERK, p-ERK, p38 and p-p38 were normalized based on the internal control β-actin. **C**. Expression of PKC-α and PKC-β1,cells were pretreated with vehicle or curcumin at the indicated concentration (5–50 μM) for 1 h, followed by PMA for 48 h. **D**. Densitometry measurements of protein analysis. The mean density values of PKC-αand PKC-β1 are expressed as ratios relative to that of β-actin. *P < 0.05 vs PMA group, **P < 0.05 vs CTL group.

**Figure 4:**
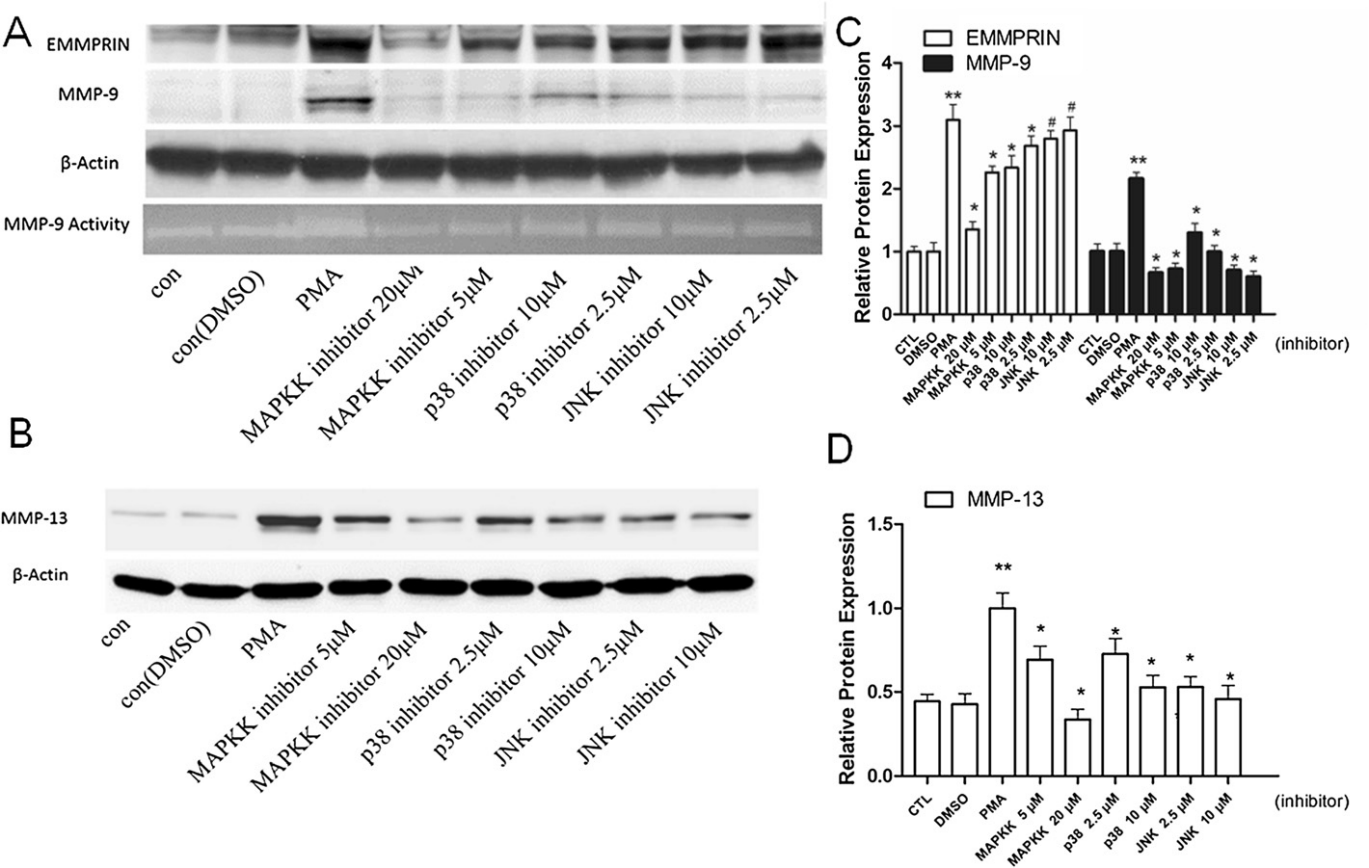
**PMA induced EMMPRIN, MMP-9 and MMP13 expression by macrophages is blocked by a specific ERK, p38 and/or JNK inhibitors. A**-**B**. The addition of a specific ERK inhibitor (PD98059, 5 and 20 μM), p38 inhibitor (SB203580, 2.5 and 10 μM) completely blocks PMA-induced MMP-9 protein expression and activation, and p38-specific inhibitor showed weaker function. ERK1/2 and p38-specific inhibitor inhibitor significantly decreased EMMPRIN expression, whereas JNK specific inhibitor showed no inhibitory effect. **C**-**D**. ERK1/2, p38 and JNK-specific inhibitor at high dose showed remarkable inhibitory effect on MMP-13 expression. *P < 0.05 vs PMA group, #*P* >0.05 vs PMA group.

Previous studies from other groups and our group indicate that PMA promotes the level of EMMPRIN and MMP-9 through activating MAPK signaling pathways [8,29,30]. PMA also is a strong inducer of protein kinase C, pkc signal paly a role during PMA induced cell differentiation and adhension [31,32]. Thus, we wondered whether the reduced EMMPRIN expression was through the MAPK or PKC pathway. To test this hypothesis, THP-1 cells were first pretreated with curcumin for 1 hour before incubating with PMA for another 48 hours. Western data showed that curcumin significantly inhibited the phosphorylation of ERK1/2, p38 MAPK, JNK and PKCα, PKCβ1 induced by PMA (Figure 3A-B). To further explore which MAPK signaling involved in the upregulation of MMP-9, MMP13 and EMMPRIN in PMA induces THP-1 cell. We next examine the expression of them after treated with ERK1/2-specific inhibitor (PD98059), p38-specific inhibitor (SB203580), and JNK-specific inhibitor (SP600125).As shown in Figure 4, ERK1/2 and JNK-specific inhibitor significantly downregulated MMP-9 expression, and activation ,and p38-specific inhibitor showed weaker function. ERK1/2 and p38-specific inhibitor inhibitor significantly decreased EMMPRIN expression, whereas JNK specific inhibitor showed no inhibitory effect (Figure 4A,C). For MMP-13, ERK1/2, p38 and JNK-specific inhibitor at high dose showed remarkable inhibitory effect(Figure 4B,D). In conclusion, our result suggest that MAPK signaling and PKC pathways are involved in the regulation of EMMPRIN, MMP-9 and MMP-13 expression.

### Curcumin suppresses MMP-9 and EMMPRIN expression by inhibiting phosphorylation of AMPK through MAPK pathways

**Figure 5:**
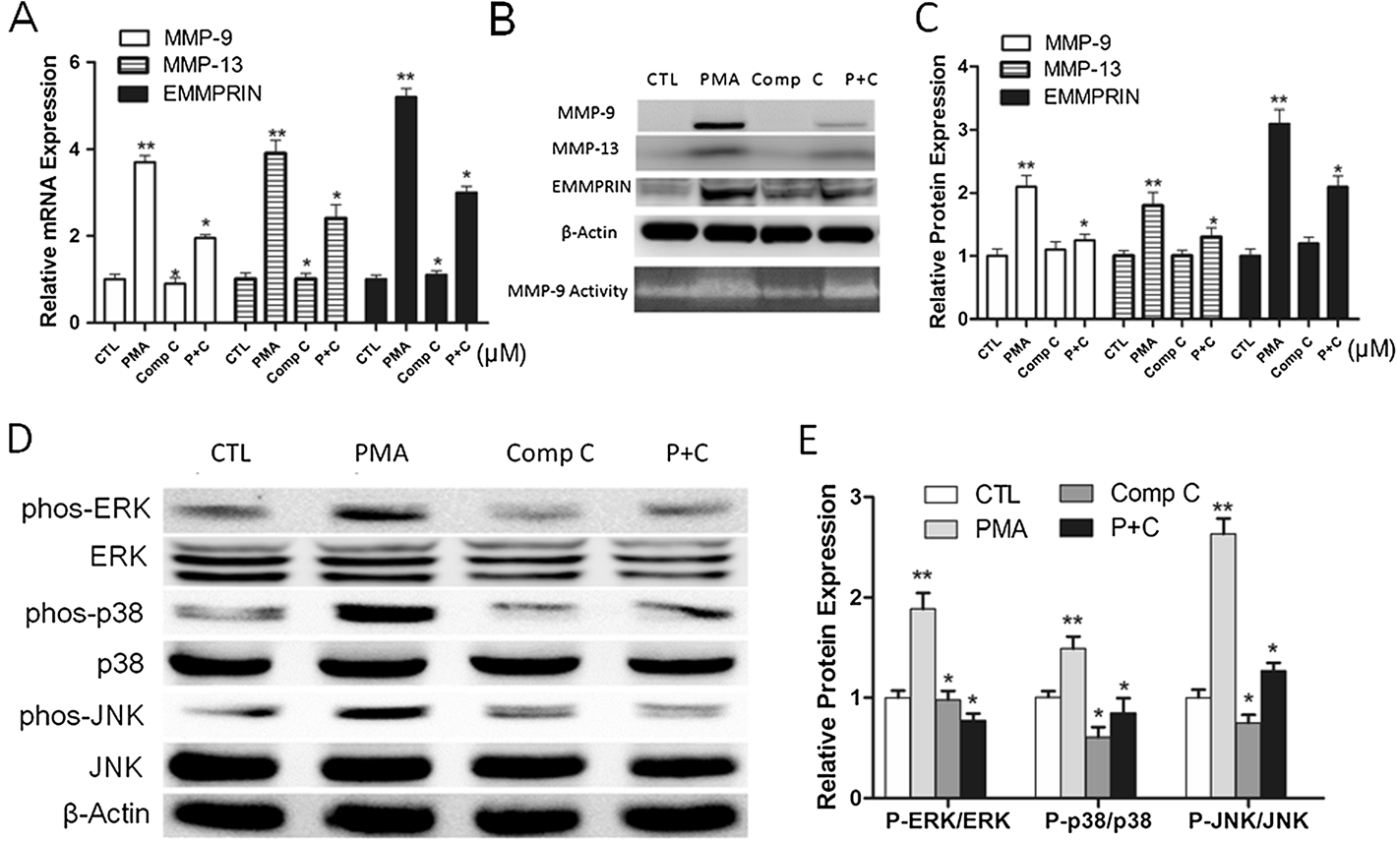
**AMPK inhibitor mediated EMMPRIN, MMP-9 and MMP13 expression inhibition depends on the activation of MAPK pathway. A**-**C**. Compound C, AMPK inhibitor, significantly inhibits EMMPRIN, MMP-9 and MMP13 expression in PMA induced THP-1 cells. Cells were pretreated with vehicle or Compound C (10 μM) for 1 h, followed by PMA for 48 h. The mRNA level of EMMPRIN, MMP-9 and MMP13 was determined by qPCR **(A)**, and protein level was determined by Western blot **(B)** and quantified by densitometric analysis **(C)**. Comp C indicates group treated with compound C; P + C indicates group treated with both PMA and compound C. **D**-**E**. Compound C inhibited the activation of MAPK pathway. Differentiated THP-1 cells were treated with indicated agents, and assayed by Western blot using indicated antibodies **(D)** and quantified by densitometric analysis **(E)**. *P < 0.05 vs PMA group, **P < 0.05 vs CTL group.

To further elucidate whether AMPK has an effect on MAPK pathway after cells exposed to curcumin, we first determine whether AMPK inactivation promotes MMP-9, MMP-13 and EMMPRIN expression. As shown in Figure 5A-C, inhibition AMPK by compound C (AMPK inhibitor) dramatically suppressed MMP-9, MMP-13 and EMMPRIN expression, indicating that AMPK chronic activation are important for PMA induced MMP-9, MMP-13 and EMMPRIN expression. Thus, inhibiting the activation of AMPK by curcumin (Figure 2C) may also contribute to attenuated MMP-9, MMP-13 and EMMPRIN expression. In addition, compound C also reduced the phosphorylation of p38, JNK, and ERK in PMA induced THP-1 cells (Figure 4D-E), suggesting that the AMPK inhibitor diminished the activation of p38, JNK, and ERK pathways. Taken together, we concluded that curcumin significantly inhibited phosphorylation AMPK through MAPK pathways in dose-dependent manner, which led to down-regulated EMMPRIN and MMP-9 expression in PMA-induced THP-1 cells.

## Discussion

In this study, our data support a novel effect of curcumin on the expression level of EMMPRIN, MMP-9 and MMP-13, suggesting that curcumin could be a potential therapeutic agent for ameliorating the development of atherosclerosis plaque. We found that curcumin inhibits EMMPRIN MMP-9 and MMP-13, expression via PKC and AMPK-dependent pathway in PMA induced THP-1 cells. Elevated expression and activity of MMP-13, MMP-9 and EMMPRIN are correlated with advanced atherosclerotic lesions followed by plaque rupture and myocardial infarction [8,19,33,34],which can be inhibited by curcumin.

To elucidate the molecular mechanisms underlying anti-atherolsclerosis activity of curcumin in PMA induced THP-1 cells, we first measured the protein level of phosphorylated AMPKα in THP-1 differentiated macrophage. AMPK, the master regulator of energy metabolism, emerges as a kinase that controls glycogen utilization, lipid metabolism, fatty acid uptake and oxidation, and protein synthesis [35,36]. AMPK is also necessary for the invasive ability, the MMP-9 activity of THP-1 cells [37,38], and PMA induced THP-1 cell adhesion to endothelial cells [39]. PMA has been shown to induce the activation of AMPKα, and the inactivation of AMPKα resulted in down-regulation of MMP-9, MMP-13 and EMMPRIN. As reported previously, Curcumin was shown to inhibit the activation of AMPKα, although other research demonstrated different result [40,41]. The discrepancy may be due to different cell type and/or different inducing condition. However, no study has determined the role of curcumin in the long term activation of AMPKα. In our study, we found that AMPK is activated during 48 h PMA induced cell differentiation, and curcumin suppresses the chronic activation of AMPKα in a dose-dependent manner. Consistent with our data, the activation of AMPKs has been reported to induce cell differentiation, including bone marrow-derived cells differentiation into endothelial cells [42] and osteoblastic differentiation [43]. In addition, we observed that compound C (AMPK inhibitor) inhibits MMP-9, MMP-13 and EMMPRIN expression level in PMA induced THP-1 cell differentiation. PKC signal were actived during PMA induced cell differentiation and adhesion [31,32]. Our found showed PKC was actived by in PMA induced THP-1 cells, curcumin can inhibit the activation of PKCα and PKCβ1. Therefore, through inactivating AMPKs and PKC, curcumin decreases the MMP-9, MMP-13 and EMMPRIN level which results in inhibiting monocyte/macrophage differentiation.

In addition, compound C also suppress the phosphorylation of three major classes of MAP kinase signaling (ERK, JNK, and p38), suggesting that curcumin may suppress MMP-9, MMP-13 and EMMPRIN level by inactivation of MAPK pathways. Previous data indicate that EMMPRIN and MMPs can be regulated by different factors, especially in MAPK pathways. For example, Lee et al. reported that MMP-9 production was enhanced in murine macrophages via activation of ERK and p38 MAPK [44,45]. Moreover, MMP-9, MMP13 and EMMPRIN level can be suppressed by ERK inhibitors or JNK siRNA [3,46,47]. Consistent with our previous studies [28,29], MAPK cascades (p38, ERK1/2, and JNK) are activated to induce the expression of MMP-9 [48-50], MMP13 [51,52] and EMPRIN [53,54].

As shown in this study, PMA induced the phosphorylation of ERK1/2, p38 and JNK. Curcumin inhibits MAPKs phosphorylation, which contributes to the down-regulation of MMP-9, MMP-13 and EMMPRIN expression. This was further supported by the finding that the specific inhibitor of ERK1/2, p38 and JNK showed different extent in PMA induced protein expression. Similarly, we found that compound C suppresses the phosphorylation of ERK1/2, p38 and JNK, and the expression of MMP-9 and EMMPRIN. All these results suggest that curcumin suppresses the activation of ERK1/2, p38 and JNK by inhibiting p-AMPK and PKC.

## Conclusion

**Figure 6:**
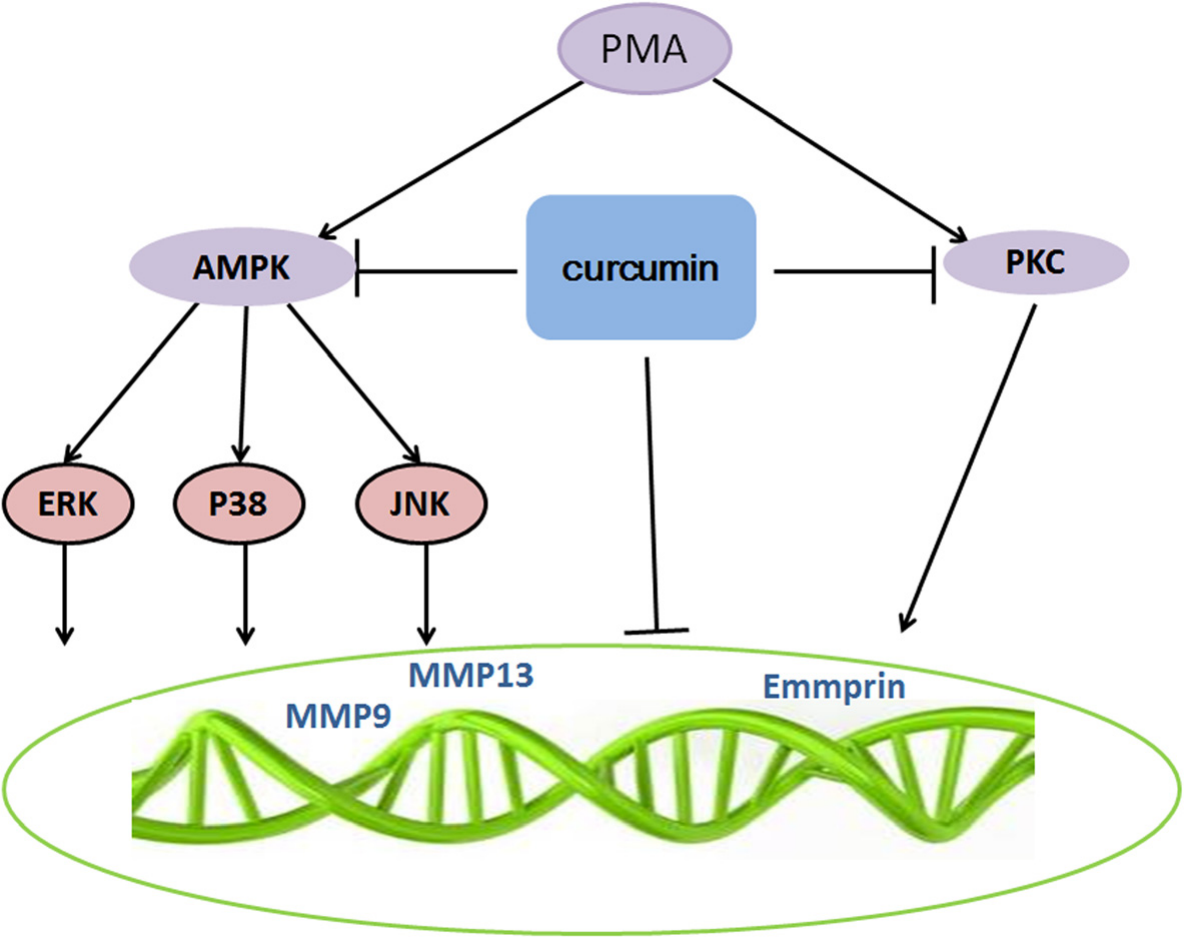
**Model for regulation of MMP-9, MMP-13 and EMMPRIN by curcumin.** PMA induced EMMPRIN, MMP-9 and MMP13 expression by macrophages through AMPK and PKC pathway.Curcumin attenuates expression of MMP-9, MMP-13 and EMMPRIN by inhibiting the activation of AMPK and PKC pathway.

In summary, we showed that curcumin attenuates MMP-9, MMP-13 and EMMPRIN expression through the down-regulation of the AMPK and PKC pathway. (Figure 6). Moreover, we identified AMPK as a novel negative regulator of MMP-9 and EMMPRIN expression in THP-1 cell during differentiation. We also indicate that AMPK - MAPK and PKC pathways are involved in inhibiting MMP-9, MMP-13 and EMMPRIN expression. Because MMP-9 and MMP-13 plays an important role in the rupture of atheromatous plaques, our findings shed novel insight into the regulatory mechanism of MMP-9 and MMP-13 expression, the function of AMPK, and a potential treatment of atherosclerosis by curcumin.
